# Treatment of Oil Wastewater and Electricity Generation by Integrating Constructed Wetland with Microbial Fuel Cell

**DOI:** 10.3390/ma9110885

**Published:** 2016-11-01

**Authors:** Qiao Yang, Zhenxing Wu, Lifen Liu, Fengxiang Zhang, Shengna Liang

**Affiliations:** 1 School of Food and Environment, Dalian University of Technology, Panjin 124221, China; wzx162@mail.dlut.edu.cn (Z.W.); lifenliu@dlut.edu.cn (L.L.); fengxiang@mail.dlut.edu.cn (F.Z.); lsnlg@mail.dlut.edu.cn (S.L.); 2 Key Lab of Industrial Ecology and Environmental Engineering (Ministry of Education), Dalian University of Technology, Dalian 116024, China

**Keywords:** oil wastewater, constructed wetland, microbial fuel cell, wastewater treatment, power density

## Abstract

Conventional oil sewage treatment methods can achieve satisfactory removal efficiency, but energy consumption problems during the process of oil sewage treatment are worth attention. The integration of a constructed wetland reactor and a microbial fuel cell reactor (CW-MFC) to treat oil-contaminated wastewater, compared with a microbial fuel cell reactor (MFC) alone and a constructed wetland reactor (CW) alone, was explored in this research. Performances of the three reactors including chemical oxygen demand (COD), oil removal, and output voltage generation were continuously monitored. The COD removals of three reactors were between 73% and 75%, and oil removals were over 95.7%. Compared with MFC, the CW-MFC with a MnO_2_ modified cathode produced higher power density and output voltage. Maximum power densities of CW-MFC and MFC were 3868 mW/m^3^ (102 mW/m^2^) and 3044 mW/m^3^ (80 mW/m^2^), respectively. The plants in CW-MFC play a positive role for reactor cathode potential. Both plants and cathode modification can improve reactor performance of electricity generation.

## 1. Introduction

Oil-contaminated wastewater occupies about 90% of the total sewage in the exploitation of oil fields. Due to the complexity of wastewater ingredients, such as the large amount of petroleum composition, the high concentration of salt ions, organic matter, and other impurities, oil sewage is a serious threat to environmental security. The traditional oil sewage treatment process can be summarized as oil separation, flotation degreasing, and filtering, which consume an enormous amount of energy, with an energy consumption by electroflotation of 0.67 kWh/m^3^ [1]. Wetland technology utilizes microbial biochemistry function for sewage purification, which is simple, strong adaptability and the operation cost is lower than other processing methods. There are shortcomings, such as the large occupied area and the long processing time. Microbial fuel cell reactors (MFCs) provide opportunities for the sustainable production of energy by biodegradable compounds [2]. MFCs have been widely verified using different pollutants such as domestic wastewater, brewery wastewater, and starch wastewater [3,4,5]. When MFC technology is used to treat wastewater, chemical bond energy stored in wastewater can be regained, and energy consumption is reduced compared with the traditional processing procedure [6]. Constructed wetland reactors integrated with microbial fuel cell reactors (CW-MFCs) constitute equipment with a subtle combination of constructed wetlands and microbial fuel cells, in which electricity generation can be enhanced on account of the rhizosphere effect of wetland plants, and pollutants in the wastewater are effectively removed due to the synergistic effect of the two units [7]. Both constructed wetland and microbial fuel cells comprise an aerobic and anaerobic zone based on a dissolved oxygen concentration, and reduction and oxidation may take place in both reactors. These similarities form the basis of the combination of two units [8]. CW-MFC have been applied to treat azo dye wastewater. The experimental results showed that, after 96 h, the decolorizing rate reached 76.2%, 80.87%, 69.29%, and 80.87% when the initial concentration was 2000, 1500, 1000, and 500 mg/L, respectively. The highest power density of 15.73 mW/m^2^ and maximum current density of 69.75 mA/m^2^ was obtained at an initial concentration of 1000 mg/L [9]. There has been little report of CW-MFCs used in oil sewage treatment.

In this study, three reactors were constructed—CW-MFC (with plant roots placed at the cathodic zone and a connected external circuit), MFC (with no plant), and CW (with a disconnected external circuit). These three systems were then compared in terms of power generation and pollutant removal.

## 2. Materials and Methods

### 2.1. Reactor Configuration

**Figure 1:**
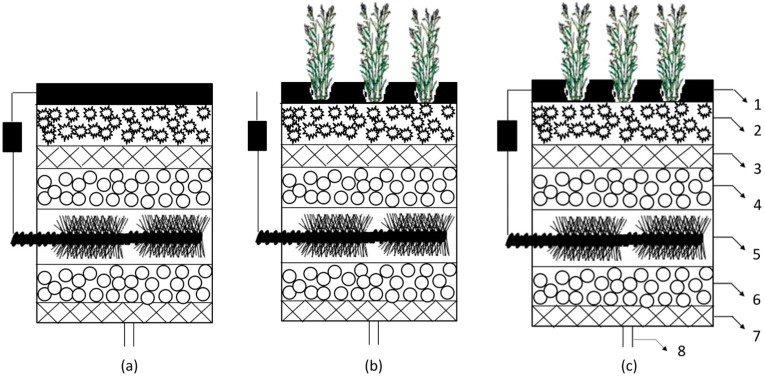
Schematic of reactors MFC (**a**); CW (**b**); CW-MFC (**c**): (1) Modified carbon felt the cathode. (2) Activated carbon. (3) Glass wool. (4) Gravel. (5) Carbon brush anode. (6) Gravel. (7) Glass wool.

The CW, MFC, and CW-MFC reactors were built using a polyacrylic plastic column (250 mm × 110 mm diameter). Crushed stone, glass wool, and activated carbon were used as filler materials as shown in Figure 1. The anode was installed at the lower part of the cylinder, and the anode effective volume was 0.25 L. Volume power density was calculated based on this value. The cathode was installed on the top of the cylinder with the same surface area of 95 cm^2^, which was used to calculate area power density. One 30 mm × 4 mm diameter inlet pipe was equipped at the bottom of the cylinder, and influent was pumped into the reactor at a rate of 17 mL/h (hydraulic retention time was three days). Effluent overflowed from the top of the reactor. *Acorus calamus* were utilized as the plants of CW, with their roots placed under the cathode layer. The total volume of the reactors was 2.4 L with a net volume (liquid volume) of 1.2 L. 

### 2.2. Electrode Materials

**Figure 2:**
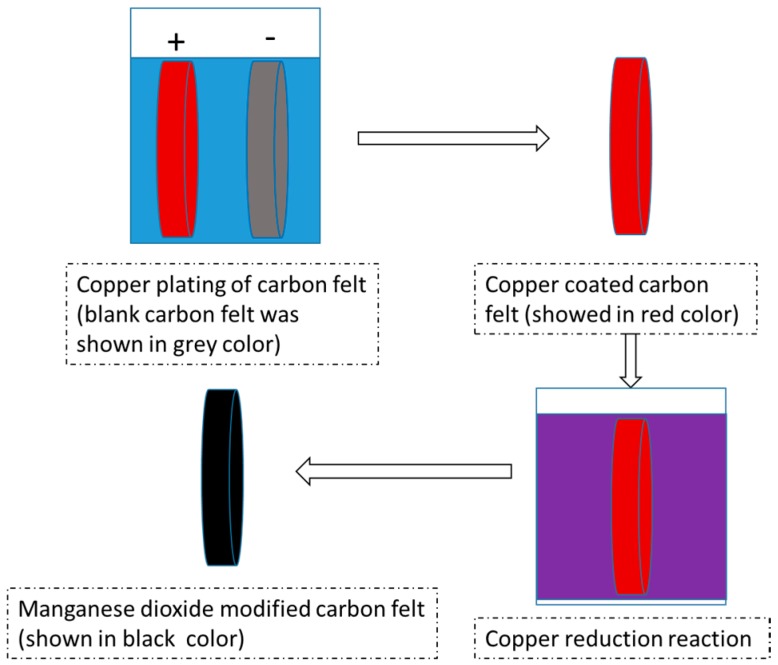
The procedure of manganese dioxide modification of blank carbon felt (arrows indicated the order of operations).

Carbon fiber brush after heat treatment was used as the anode material [10]. Blank carbon felt or manganese dioxide (MnO_2_)-modified carbon felt served as the cathode material. Manganese dioxide modified carbon felt was fabricated as follows: Carbon felt was cut into a fixed shape (110 mm diameter) and cleaned via dipping in acetone, ethanol, and demonized water sequentially to remove surface impurities. After drying in the air-blower-driver drying closet, carbon felt was placed in nitric acid and heated in 65 °C water bath for six hours. Copper electroplating was operated under 4 V direct current voltage (current was about 0.93 A) in a 0.5 mol/L CuSO_4_ electrolyte solution, with a positive electrode connected to the copper sheet and a negative electrode connected carbon felt. Electrode spacing was 10 mm, and electroplating time was 180 s. After both sides of the carbon felt were electroplated, the copper-plated carbon felt was soaked in an acidic potassium permanganate solution of a 65 °C water bath for 6 h to finish in situ MnO_2_ chemical vapor deposition. The process of modified carbon felt is shown in Figure 2.

### 2.3. Inoculation and Operation of Reactors

The three reactors were inoculated with the effluent of stable running MFCs. The nutrient solution contained 1 g/L sodium acetate in 50 mM phosphate buffer solutions (PBS, 0.31 g NH_4_Cl, 0.13 g KCl, 10.32 g Na_2_HPO_4_·12H_2_O, 3.32 g NaH_2_PO_4_·2H_2_O in one liter) with vitamin and trace essential elements [11,12]. When voltages increased and were kept stable for two days, reactors were considered start-up completed. The influent was changed to a mixed solution of 20% oil sewage and 80% inoculation nutrient solution until voltage was kept stable for two days. Then, this process was repeated with 40%, 60%, and 80% oil sewage. The proportion of oil sewage increased gradually until the influent was 100% oil sewage. The reactors performances were then analyzed. The three reactors were operated at room temperature. External resistances for MFC and CW-MFC were 1000 Ω during the entire start-up and operation, except the duration of the power density curves were measured.

### 2.4. Analysis Methods

Oil content in oil wastewater was determined by the weight method. Chemical oxygen demand (COD) and biochemical oxygen demand (BOD) were analyzed by the standard methods without filtration or sample pretreatment [13,14]. Total organic carbon (TOC) was automatically measured using a Multi N/C 2100 TOC Analyzer (Analytik Jena, Jena, Germany) after the sample was filtered by a 0.22 μm membrane. A modified carbon felt surface structure was analyzed using a scanning electron microscope (SEM, Hitachi, Tokyo, Japan). The output cell voltage was recorded every 1 min with a data acquisition system (MPS010-602, Altai, China) connecting with a personal computer. The power was calculated according to *P* = *V*^2^/*R*, where *P* = power, *V* = voltage, and *R* = resistance [15]. Power density (mW/m^2^ or mW/m^3^) was normalized by the surface area of the cathode (equal with CW surface area) or anode effective volume. Polarization and power curves were obtained by changing the external resistance from 5000 to 100 Ω every 30 min to confirm the measured voltage values were stable. Electrode potential was referred to the saturated calomel electrode (SCE).

## 3. Results and Discussion

### 3.1. Oil Wastewater

The oil-contaminated wastewater sample was collected from an oil wastewater treatment plant located in the city of Panjin, China. The quality of the oil wastewater was described as having a COD of 520 ± 42 mg/L, a BOD of 109 ± 3 mg/L, oil content of 235 ± 12 mg/L, and total organic carbon (TOC) of 33 ± 1 mg/L. The raw wastewater pH value and conductivity was 7.32 and 1882 μS/cm, respectively. The oil wastewater served as the influent for the three reactors during the performance test without the addition of any other nutrients.

### 3.2. Power Generation with Different Reactors

**Figure 3:**
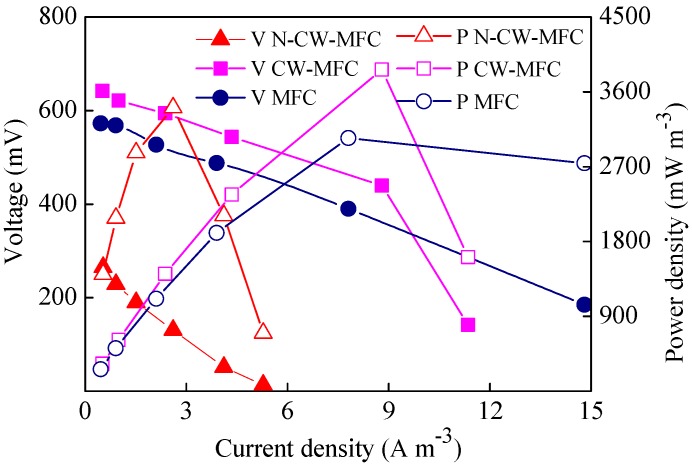
Polarization and power density curves for N-CW-MFC (constructed wetland integrated with microbial fuel cells of a blank carbon felt cathode), CW-MFC (constructed wetland integrated with microbial fuel cells of a MnO_2_-modified cathode), and MFC (microbial fuel cells of a MnO_2_-modified cathode).

After the reactor voltages were stable using oil wastewater as an influent, the electrochemical performances of the reactors were analyzed, including the polarization and power density curves. The power density of the N-CW-MFC (constructed wetland integrated with microbial fuel cells of a blank carbon felt cathode) was 3409 mW/m^3^, calculated by the anode effective volume, or 90 mW/m^2^, calculated by the cathode area (N-CW-MFC in Figure 3). It was increased to 3868 mW/m^3^ (102 mW/m^2^) when the cathode was MnO_2_-modified (CW-MFC in Figure 3). Carbon materials are commonly used in the anodes and cathodes of microbial fuel cells [16,17,18]. Compared with the anode, the cathode may play a more prominent role in reactor performance. MnO_2_, which possesses good capacitive and catalytic properties, is widely applied to capacitor materials and electrode catalyst materials. Its morphology and crystal structure influence catalytic effect. MnO_2_ has good electrochemical catalytic properties and can be generated by using copper as a reducing agent with a certain concentration in an acid potassium permanganate solution [19,20]. In this study, the maximum power density was improved by 13.5% when using a MnO_2_-modified cathode. 

**Figure 4:**
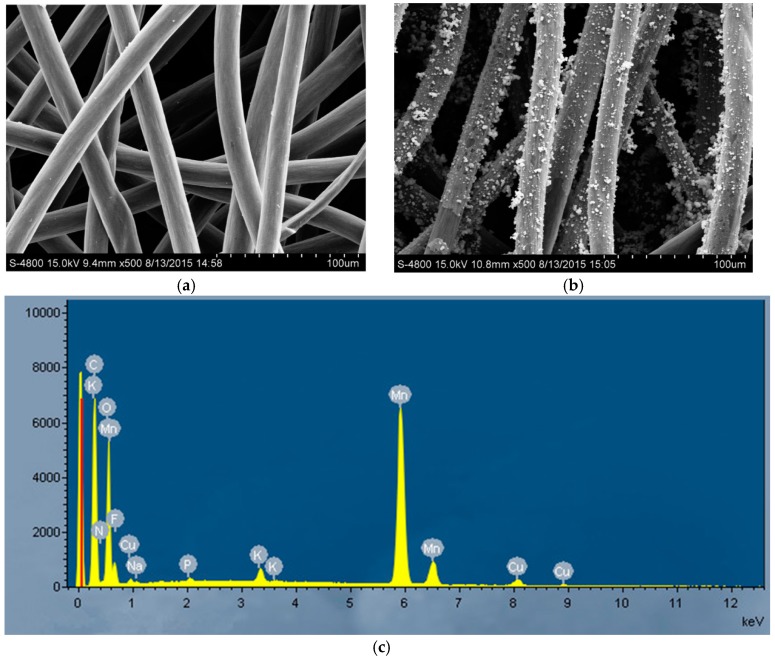
Scanning electron microscopy analysis. (**a**) Scanning electron microscopy picture of unmodified carbon felt; (**b**) Scanning electron microscopy picture of modified carbon felt; (**c**) Energy dispersive X-ray spectrum (EDS) of modified carbon felt.

Modified carbon felt and blank carbon felt were analyzed with a scanning electron microscope. Blank carbon felt has a smooth carbon surface (Figure 4a), while a large amount of small spherical material was found at the surface of the modified carbon felt (Figure 4b). Energy dispersive X-ray spectrum (EDS) analysis showed that Mn element increased significantly (Figure 4c). Therefore, a modification of the carbon felt cathode was accomplished, and the improvement of electricity generation was attributed to the MnO_2_ modification of the cathode in CW-MFC. 

The CW-MFC reactor with a modified cathode achieved a power density of 3868 mW/m^3^ or 102 mW/m^2^ (CW-MFC in Figure 3), compared with the MFC with a similar cathode (3044 mW/m^3^ or 80 mW/m^2^); power density was increased by 27.1%. This demonstrated that the plants in the CW-MFC can improve reactor performance in terms of electricity generation. The probable cause of such a phenomenon may be related to the plant roots. Oxygen reduction reaction is often considered to occur at the cathode of MFCs, described as O_2_ + 4H^+^ + 4e^−^ = 2H_2_O. This process may be enhanced by the rhizosphere effect of plants [21]. Plants can transmit oxygen generated by photosynthesis to the roots, or active microorganisms might be enriched by nearby plant roots. Either way, the cathode performance was improved in CW-MFC.

**Figure 5:**
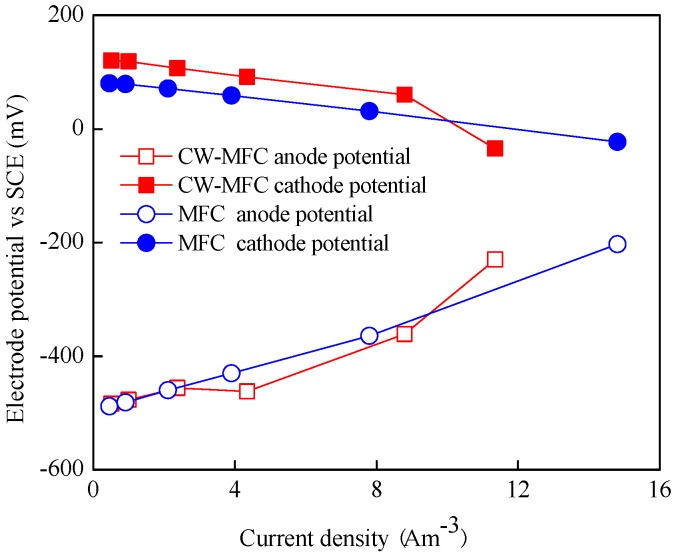
Anode and cathode potential curves.

The electrode potential curves in Figure 5 show the difference in cathode potential for MFC and CW-MFC reactors. Along with the increase in current densities, the anode potential curves were almost identical with each other. The cathode potential of CW-MFC was more positive than that of MFC. In detail, the open circuit potential of the CW-MFC cathode and the MFC cathode was 120 and 80 mV, respectively. The open circuit potential of the CW-MFC cathode was 49.6% higher than MFC. The difference in cathode potential may prove that the plant played a positive role for cathode performance in the CW-MFC reactor.

### 3.3. Pollution Removal Performance

**Figure 6:**
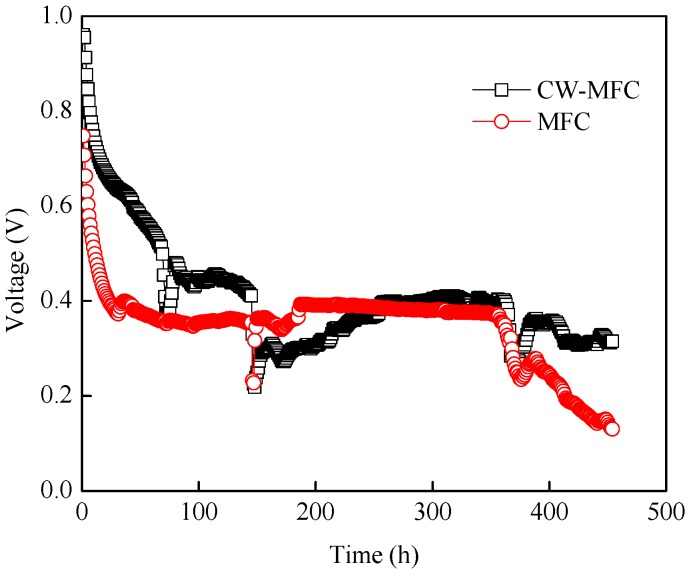
Voltage variation after new modified cathodes were placed in reactors. Triple sampling was operated during the period of 200–350 h.

After the cathodes were replaced by newly modified carbon felt, CW, MFC, and CW-MFC operated continuously at 1000 Ω resistance. Oil sewage was pumped into the reactor through the injection port with a hydraulic retention time of 3 days. Output voltage was recorded and is presented in Figure 6. The highest voltages of 990 (CW-MFC) and 760 mV (MFC) were achieved at the beginning. Voltage dropped gradually along with time and then stabilized at about 430 (CW-MFC) and 400 mV (MFC) under 1000 Ω resistors. The corresponding area power density was 19 and 17 mW/m^2^, and power density normalized by the volume was 740 and 640 mW/m^3^. The precipitous drop at 150 h for CW-MFC was caused by the accidental leakage of reactor. After about 360 h, both CW-MFC and MFC voltages began to decrease. It was speculated that MnO_2_ was reduced to manganese ion gradually, as the described reaction of MnO_2(S)_ + 4H^+^ + 2e^−^ = Mn^2+^ + 2H_2_O [22]. Along with the disappearance of original manganese dioxide, cathode catalytic performance decreased, and voltage dropped. The detail of this hypothesis will be further studied in future research. During the stable running duration of these reactors, TOC removals were 59% ± 1% (MFC), 48% ± 1% (CW), and 57% ± 1% (CW-MFC), while COD removals were 75% ± 6% (MFC), 73% ± 5% (CW), and 73% ± 6% (CW-MFC). COD removal was contributed by microbes in the system, where microbes oxidized the organic substrate as carbon sources to support its own activity or to generate electricity [23]. No oil content was detected from the effluent of CW, MFC, or W-MFC using the weight method, which is a commonly used analysis method as it is not affected by oil variety. The oil content of effluent could be considered lower than 10 mg/L as the limit of detection was 10 mg/L, so oil removal was calculated as over 95.7%.

## 4. Conclusions

This study showed the difference between three reactors with respect to oil wastewater treatment and electricity generation. A CW-MFC with a modified cathode produces the highest power density, 3868 mW/m^3^, which is higher 27.1% than that of a MFC and 13.5% than that of a N-CW-MFC with an unmodified cathode. Plants in the CW-MFC played a positive role for reactor cathode potential. CW-MFC and MFC voltages were basically stable at 430 and 400 mV, respectively. All three reactors had satisfactory effects on oil removal of over 95.7%. The COD removal rate was from 73% to 75%.
